# Progression and Regression of Hepatic Lesions in a Mouse Model of NASH Induced by Dietary Intervention and Its Implications in Pharmacotherapy

**DOI:** 10.3389/fphar.2018.00410

**Published:** 2018-05-01

**Authors:** Zhi-Ming Ding, Yue Xiao, Xikun Wu, Haixia Zou, Shurong Yang, Yiyun Shen, Juehua Xu, Heather C. Workman, Amy L. Usborne, Haiqing Hua

**Affiliations:** ^1^Lilly China R&D Center, Shanghai, China; ^2^Covance Inc., Indianapolis, IN, United States; ^3^Lilly Research Laboratories, Eli Lilly and Co., Indianapolis, IN, United States

**Keywords:** non-alcoholic steatohepatitis, steatosis, inflammation, fibrosis, obeticholic acid, CCR2/5, pathogenesis

## Abstract

Understanding of the temporal changes of hepatic lesions in the progression and regression of non-alcoholic steatohepatitis (NASH) is vital to elucidation of the pathogenesis of NASH, and critical to the development of a strategy for NASH pharmacotherapy. There are challenges in studying hepatic lesion progression and regression in NASH patients due to the slow development of NASH in humans, one being the requirement for multiple biopsies during the longitudinal follow-up. Here we studied lesion progression and regression in the diet-induced animal model of NASH by application or removal of the pathogenic diet for multiple time periods. Male C57BL/6 mice fed Western diet developed progressive hepatic steatosis/macrovesicular vacuolation, inflammation, and hepatocyte degeneration, as well as perisinusoidal fibrosis and occasionally portal fibrosis as early as 2 months after initiation of the Western diet. In the same period, the mice exhibited elevated ALT (alanine aminotransferase) and AST (aspartate aminotransferase) enzyme activities, CK18 (cytokeratin−18), PIIINP (N-terminal propeptide of type III collagen), and TIMP-1 (tissue inhibitor of metalloproteinase−1). Hepatic steatosis diminished rapidly when the Western diet was replaced by normal rodent chow diet and hepatic inflammation and hepatocyte degeneration were also reduced. Interestingly, perisinusoidal fibrosis and portal fibrosis regressed 8 months after chow diet replacement. To understand pharmacotherapy for NASH, mice with established NASH hepatic lesions were treated with either FXR agonist obeticholic acid (Ocaliva), or CCR2/5 antagonist Cenicriviroc. Similar to the diet replacement, metabolic modulator Ocaliva markedly reduced steatosis/macrovesicular vacuolation, hepatic inflammation, and hepatocyte degeneration effectively, but exhibited no significant effect on liver fibrosis. Anti-inflammation drug Cenicriviroc, on the other hand, markedly decreased inflammation and hepatocyte degeneration, and mildly decreased liver fibrosis, but exhibited no effect on hepatic steatosis/macrovesicular vacuolation. In conclusion, we found the progression of NASH hepatic steatosis/macrovesicular vacuolation, and inflammation eventually lead to hepatocyte death and fibrosis. Life style change and current pharmacotherapies in development may be effective in treating NASH, but their effects on NASH–induced fibrosis may be mild. Since fibrosis is known to be an independent risk for decompensated cirrhosis, cardiovascular events, and mortality, our study suggests that effective anti-fibrosis therapy should be an essential component of the combined pharmacotherapy for advanced NASH.

## Introduction

Development of an effective therapy for non-alcoholic steatohepatitis (NASH) relies on knowledge of NASH pathogenesis. Through longitudinal follow-up and paired liver biopsy (Singh et al., 2015; Calzadilla Bertot and Adams, 2016; Marengo et al., 2016), there is understanding of the lesion progression in NASH. Non-alcoholic fatty liver disease (NAFLD), once considered a relatively benign disease, is found to progress to NASH in a subgroup of patients. The NASH patients are characterized by hepatic inflammation, hepatocyte ballooning in addition to steatosis. Following this active phase of the disease, most patients develop perisinusoidal (zone 3) fibrosis (Brunt, 2009). Some of those patients rapidly develop more advanced portal and bridging fibrosis, which eventually lead to cirrhosis. In a small portion of those patients there may be progression to hepatocellular carcinoma (HCC) (Bugianesi et al., 2002). Although advances have been made in understanding the processes of NASH lesion progression, it is still unknown why most NAFLD patients do not eventually develop NASH, and why some NAFLD patients progress to NASH and cirrhosis at a more rapid pace (Calzadilla Bertot and Adams, 2016).

NASH lesion regression and resolution, as NASH lesion progression, is an essential part of NASH pathophysiology and is critical for the development of NASH therapeutic strategy. The observations that hepatitis B and C patients achieved sustained virological response (SVR) after interferon or other antiviral therapies exhibit regression of liver fibrosis and cirrhosis (van Zonneveld et al., 2006; George et al., 2009; Rockey, 2016) led to a conceptual revolution in hepatology, indicating that liver fibrosis and cirrhosis, once considered terminal lesions, are now possibly reversible and treatable. Currently, there is no drug officially approved for the treatment of NASH. Although Ocaliva (OCA) (Neuschwander-Tetri et al., 2015), Cenicriviroc (CVC) (Friedman et al., 2016), and Elafiranor (Ratziu et al., 2016) showed benefits in phase 2b clinical trials, their long-term benefits and safety remain to be demonstrated. Therefore, it is impossible in the near term to perform similar trials to study NASH lesion regression. Efforts have been made to study NASH lesion regression and resolution by life style changes including calorie restriction, body weight loss and physical exercise (Vilar-Gomez et al., 2015; Mahady and George, 2016). Most life style intervention studies demonstrated improvements in liver enzymes including AST and AST, or reduced tissue-based hepatic lipids. A few of those studies even showed improvement in the histopathological features of NASH (Promrat et al., 2010; Vilar-Gomez et al., 2015). In these studies, hepatic NASH lesions in male C57BL/6 mice were induced by feeding them the Western diet enriched in trans-fat, cholesterol, and fructose. After establishment of NASH lesions, the Western diet was replaced by normal rodent chow or the NASH mice were treated by Ocaliva and Cenicriviroc. Regression and resolution of hepatic NASH lesions were then monitored over time.

## Materials and methods

### Animals husbandry

Male C57BL/6 were purchased from Nanjing Biomedical Research Institute. They were housed in microisolator cages within a barrier facility at 22–24°C on a fixed 12-h light and 12-h dark cycle and were provided *ad libitum* acidified water and Purina rodent chow No. 5001 (Purina, St Louis, MO). All studies were performed at the vivarium facility of Covance, Greenfield (Indianapolis, USA) or Chem Partner (Shanghai, China) and in strict accordance with the recommendations in the Guide for the Care and Use of Laboratory Animals of the National Institutes of Health. The protocols for all studies were approved by the Covance Greenfield or Chem Partner Institutional Animal Care and Use Committee.

### NASH lesion induction and dietary intervention

NASH hepatic lesions were induced according to the protocol published by Clapper et al. (2013). Briefly 3 week old mice were fed the Western diet D09100301 (Research Diets Inc., New Brunswick, NJ) containing 40% fat and 25% fructose in calorie, and 2% cholesterol (g/g). During the lesion induction phase, their body weight, food intake, plasma lipids, glucose, and insulin were measured periodically. NASH biomarkers cytokeratin 18 (CK-18), N-terminal propeptide of type III collagen (PIIINP), and metallapeptidase I inhibitor (TIMP-1) were analyzed. At specified time points, the mice were euthanized, with blood and liver collected per protocol for analysis. After 240 days, Western diet was removed, and replaced by Teklad Global diet (Rodent, 2014) in the remaining mice (recovery group). At specified time points after the dietary change, the mice were euthanized with blood and liver collected for analysis.

### Bioanalysis

Glucose was measured uing glucometer (Roche Accu-Chek). Insulin was measured per manufacturer's instruction with a kit (Meso Scale Discovery, Cat. No. K152BZC). Serum Alanine aminotransferase (ALT), Aspartate aminotransferase (AST), triglycerides and cholesterol were analyzed by an automatic biochemical analyzer using kits provided by the manufacturer (Roche Cobas 6000). For ALT and AST detection, UV test method was optimized according to a standard method with pyridoxal phosphate activation. For cholesterol, the enzymatic colorimetric method was optimized using cholesterol esteras/oxidase. CK-18 (Cusabio, Cat. No. CSB-E14265m), PIIINP (Antibodies-online, Cat. No. ABIN367685), and TIMP-1 (R&D Systems, Cat. No. MTM100) were analyzed using ELISA kits according to the protocols provided by the manufacturers. To measure tissue-based hepatic lipids, 50 mg of fresh or frozen liver tissue was suspended in 0.5 ml of lysing buffer (140 mM NaCl, 50 mM Tris pH 7.4, 20% triton-X100), and homogenized using a polytron. Twenty microliter of tissue lysate was incubated with 0.5% deoxychloate at 37°C for 10 min. Two hundred microliter of Infinity™ Triglycerides Liquid Stabilizing Reagent (Thermo Fisher Scientific, TR22421) was added to the mixture. The final solution was incubated at 37°C for 15 min. Triglycerides were determined by measuring absorption at 500 nm. To analyze liver cholesterol, liver lysate was processed, and analyzed by LC-Mass Spectrometry (Agilent 6500). Plasma bilirubin was measured by Bilirubin Assay kit (Sigma, Cat. No. MAK126) according to manufacturer's protocol. To measure liver hydroxyproline, fifty milligram of fresh or frozen liver tissue were homogenized and boiled in 0.5 ml of 6 N hydrochloric acid at 95°C for 20 h. Hydrolysate was further diluted in water and acetonitrile:water (1:1) by 150-fold and was deproteinized with methanol containing 0.1% formic acid. The supernatant after centrifugation was finally analyzed by LC-Mass Spectrometry.

### Liver histopathological analysis

Left and right lateral lobes of a liver were collected and fixed immediately using 10% neutral buffered formalin. They were then embedded in paraffin, and sectioned into 0.5-micron sections. Before initiating the studies, oil red O staining was compared with H&E staining regarding pathological scoring and found that the two methods are consistent in evaluating microvesicular and macrovesicular vacuolations. Therefore, oil red O staining was not performed in later experiments. The sections from each animal were stained for hematoxylin and eosin (H&E) and Picro Sirius Red histochemical stains respectively. Microscopic assessment was conducted in mice fed the Western diet, as well as standard diet control animal that were collected at the same time points. Pathology endpoints such as macrovesicular vacuolation (steatosis), inflammation, hepatocellular degeneration, and fibrosis were characterized and quantified according to a modified NASH score system tabulated in Table 1, which is primarily based on publications of Brunt et al. (Brunt et al., 1999; Brunt, 2009).

**Table 1:** Pathological scorinng system for liver samples from non-alcoholic steatohepatitis mouse model.

		**Steatosis**			**Fibrosis**		
**Score**	**Inflammation**	**Macrovesicular vacuolation (steatosis)**	**Microvesicular vacuolation**	**Ballooning Degeneration**	**Perisinusoidal fibrosis**	**Portal fibrosis**	**Bridging fibrosis**
0	No inflammatory foci	No macrovesicular vacuoles	No microvesicular vacuoles	No degeneration	No fibrosis	No fibrosis	No fibrosis
1	1–2 foci/20x field	< 33%	< 33%	< 33%	< 33%	Expanded portal area	1 focus
2	3– < 4 foci/20x field	34–66%	34–66%	34–66%	34–66%	Periportal fibrosis	>1 focus, but no nodularity
3	>4 foci/20x field	>66%	>66%	>66%	>66%	Bridging fibrosis	Bridging fibrosis with nodular remodeling
4	N/A	N/A	N/A	N/A	N/A	Cirrhosis (nodular formation)	Cirrhosis (nodular formation)

### Statistical analysis

Continuous data was expressed as mean + standard error, and analyzed by one-way ANOVA followed by Dunnett's multiplicity test adjustment of *post-hoc* comparisons. Logarithm transformation may be applied if appropriate. For the analysis of categorical data, Raw pathological score data are shown in stacked bar graphs. Each bar within a graph is for a group and each stack within a bar is for a score value, color coded as: white = 0, light gray = 1, dark gray = 2, black = 3 and 4. Height of a stack indicates frequency of that score value observed and height of the whole bar indicates total number of observations of the group. Data from left and right lateral liver lobes are shown in left and right panels respectively. They are also pooled together and shown in a single stacked bar graph as well. Proportional Odds Model (POM) is used in pathological score data analysis. Scores from left and right lateral liver lobes of the same animal are considered as repeated measurement and evaluated by Generalized Estimating Equation (GEE). Odds Ratios (OR) on log scale of compound treatment groups to vehicle control are calculated with Dunnett's adjustment applied on *p*-values and 95% confidence intervals.

## Results

### Progressive hepatic lesions in diet-induced NASH model

**Figure 1:**
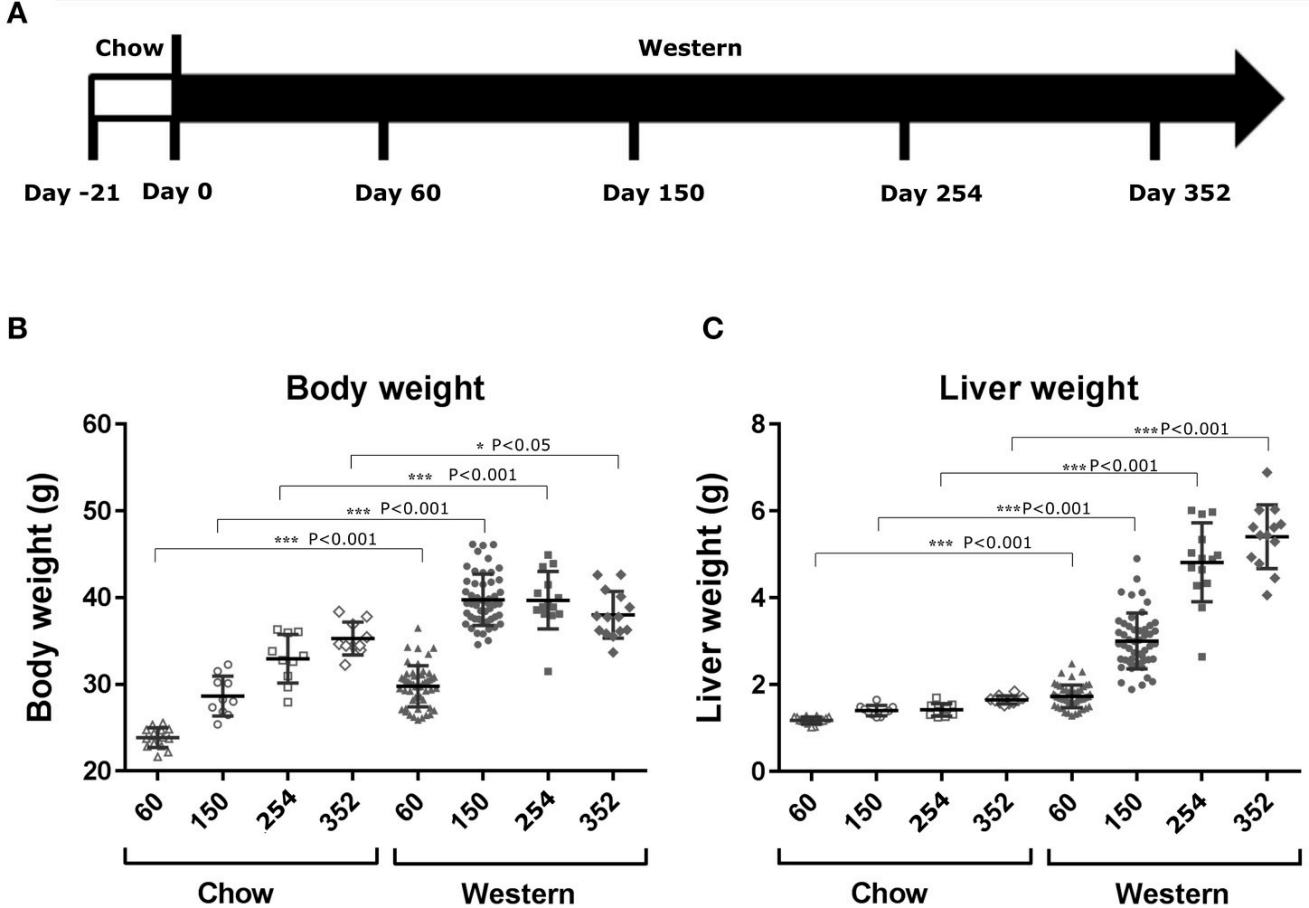
Changes in body and liver weight after the treatment with Western diet. Male C57BL/6J mice were fed Western diet as described in **(A)**. Their body weight **(B)** was recorded. Their liver weight **(C)** was measured immediately after euthanasia. Two-tail *T*-test was used to calculated *P*-values of body weight or liver weight between mice fed on chow or Western diet for the same time periods (*P < 0.05; ***P < 0.001).

**Figure 2:**
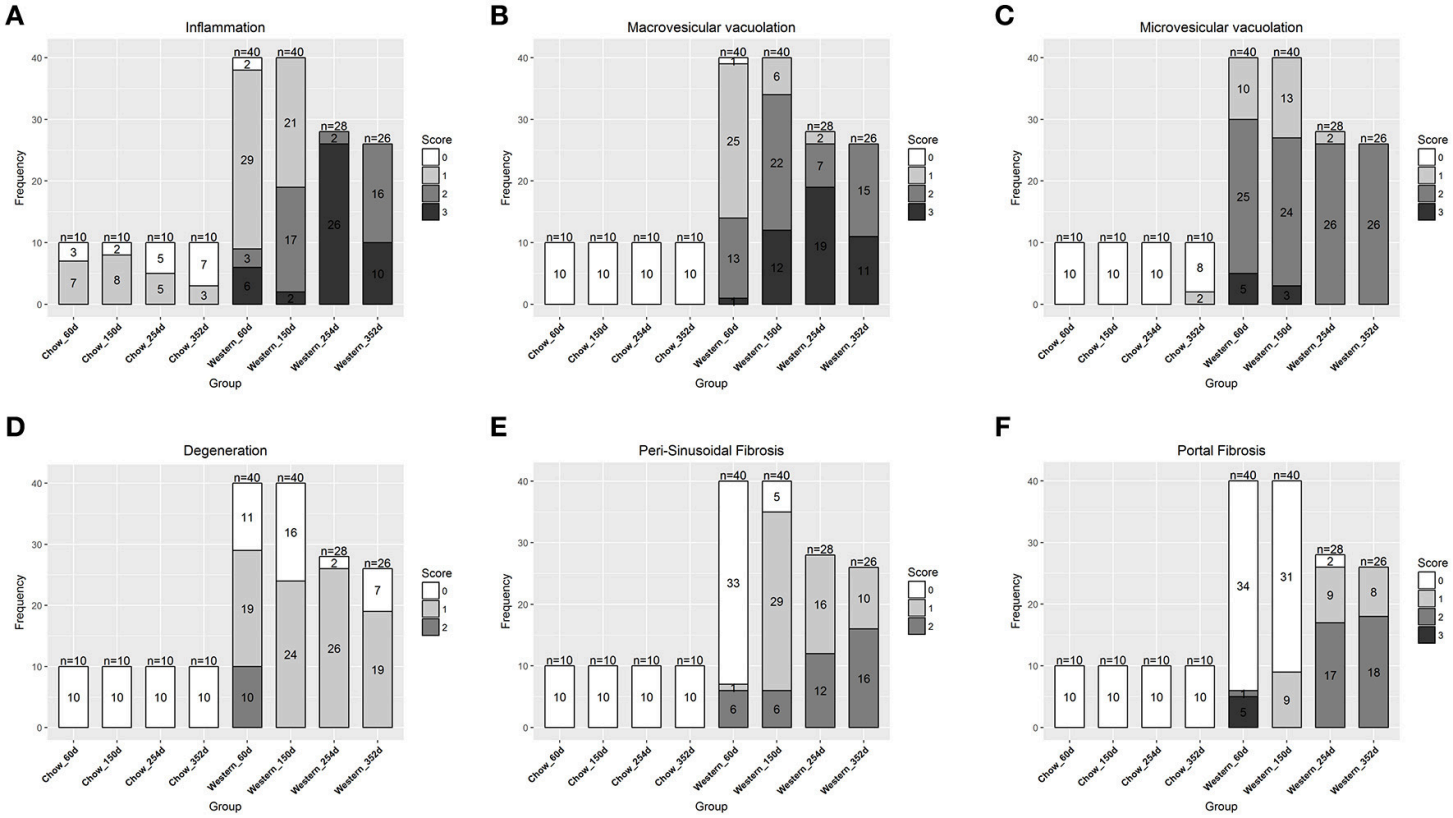
Progression of hepatic lesions in the NASH mice fed Western diet. Male C57BL/6J mice were fed chow or Western diet for different durations. The right and left lateral lobes were collected, and analyzed. The pathological scores from both lobes of an animal were pooled when performing categorical analysis. Hepatic inflammation **(A)**, macrovesicular vacuolation **(B)**, microvesicular vacuolation **(C)**, hepatocyte degeneration **(D)**, perisinusoidal fibrosis **(E)**, and portal fibrosis **(F)** were graded.

**Figure 3:**
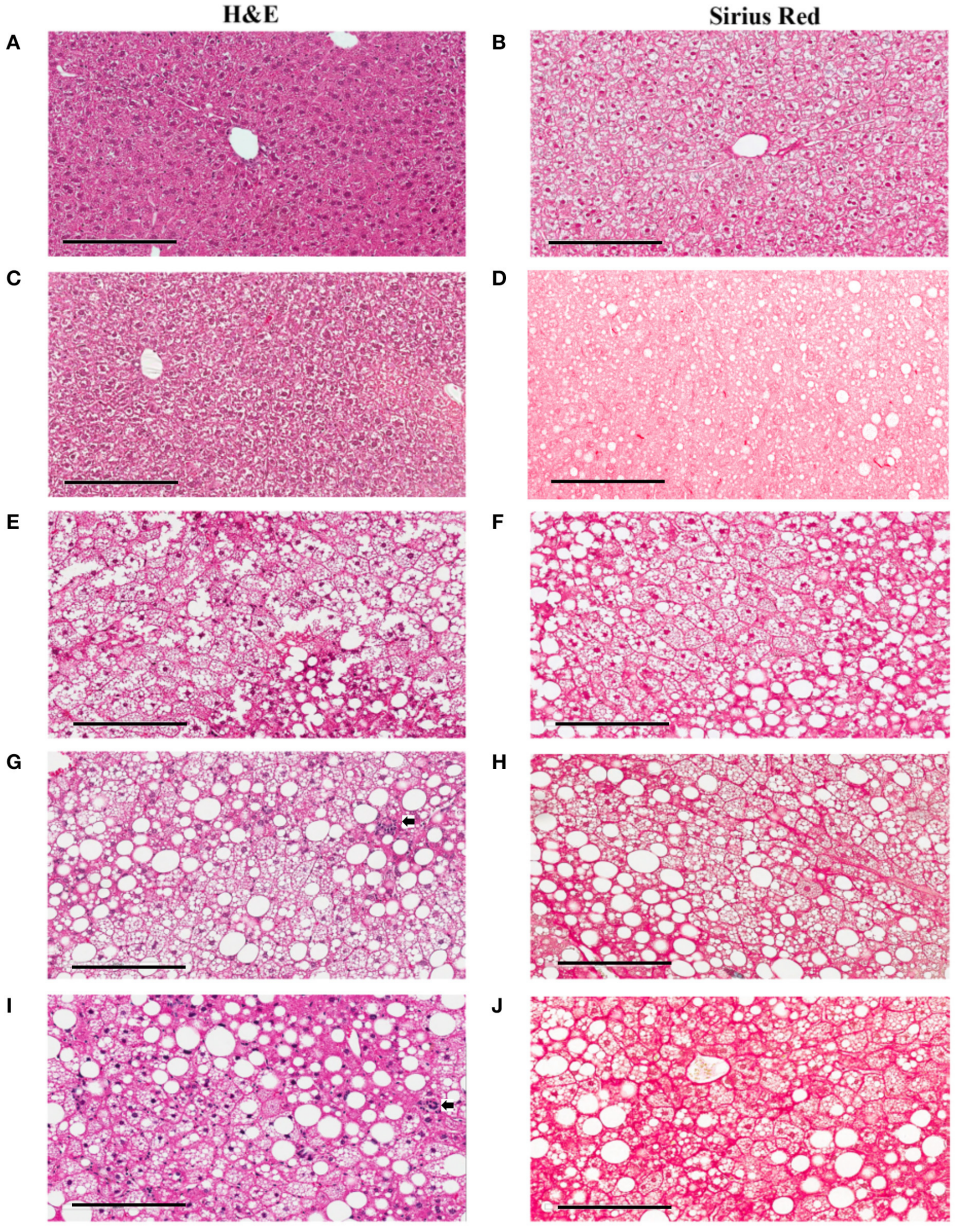
Hepatic lesions of the NASH Mice at Different Age. The representative images were taken from the liver sections of the mice fed either rodent chow **(A,B)** or Western diet for 60 **(C,D)**, 150 **(E,F)** and 245 **(G,H)**, and 352 **(I,J)** days respectively. **(A,C,E,G,I)** are the images of the liver sections stained with H&E; Panels **(B,D,F,H,J)** are the images of the liver sections stained with Sirius Red.

To study lesion progression in the diet-induced animal model of NASH, Western diet (40% fat, 24% fructose, 2% cholesterol) was fed to mice for various time periods (60, 150, 254, and 352 days) (Figure 1A). Mice fed Western diet had significantly higher body weight and liver weight (Figures 1B,C and Supplementary Figure 1). The pathology endpoints for NASH including steatosis/macrovesicular vacuolation, inflammation, degeneration, and fibrosis were quantified by board certified veterinary pathologists based on a modified NASH score system (Table 1). Compared to normal rodent chow diet fed animals (**Figures 3A,B**), Western diet treated mice had progression of inflammation and steatosis (macrovesicular and microvesicular vacuolation), inflammation and degeneration as early as 60 days (Figures 2A–D, 3E). Some mice also developed perisinusoidal and portal fibrosis (Figures 2E,F, 3C,D). When the mice were treated with Western diet, more mice developed hepatic NASH lesions with increased severity (Figures 2A–F, 3E–J, Supplementary Table 1). Blood and liver samples collected and analyzed at the same time points (Table 2) displaying hyperinsulinemia (as early as 60 days) and higher concentrations of triglyceride and cholesterol in the liver compared to normal chow vehicle controls. ALT and AST activities, CK-18, TIMP-1, and PIIINP concentrations in the plasma were significantly elevated (Table 2) compared to normal chow vehicle controls. The bilirubin and hydroxyproline levels were also elevated in western diet treated animals (Supplementary Figures 2A,B). Interestingly, unlike reports in humans, the hyaluronic acid levels in the Western diet fed animals were not increased, rather, they were moderately reduced at the 150-day time point.

**Table 2:** Biochemical analysis of blood and liver samples (Average ± SD).

**Group**	**Fed glucose (mg/dL)**	**Insulin (pg/mL)**	**Plasma TC (mg/dL)**	**Plasma TG (mg/dL)**	**Liver TC (mg/g)**	**Liver TG (mg/g)**
**PROGRESSION STUDY**						
Chow_60d	N/A	514.32 ± 191.92	79.87 ± 9.27	114.93 ± 22.32	2.21 ± 0.44	11.77 ± 1.96
Chow_150d	111.42 ± 10.13	512.43 ± 232.27	82.80 ± 13.91	135.10 ± 16.99	1.95 ± 0.03	11.74 ± 2.28
Chow_254d	127.98 ± 4.28	2156.98 ± 1048.65	77.00 ± 23.76	150.50 ± 47.86	2.14 ± 0.37	23.70 ± 10.23
Chow_352d	122.40 ± 10.73	1193.15 ± 425.73	87.90 ± 9.69	175.50 ± 49.49	2.75 ± 0.28	25.50 ± 9.88
Western_60d	N/A	1160.90 ± 755.96	129.94 ± 34.98	145.00 ± 63.77	4.07 ± 2.58	110.45 ± 107.92
Western_150d	129.85 ± 15.85	1570.33 ± 1057.65	207.34 ± 44.78	92.24 ± 28.74	26.68 ± 8.84	264.35 ± 33.81
Western_254d	127.29 ± 17.27	1261.83 ± 586.78	317.14 ± 68.07	91.79 ± 22.19	19.49 ± 4.78	274.64 ± 72.97
Western_352d	N/A	N/A	115.46 ± 18.13	684.53 ± 129.93	332.43 ± 71.93	68.86 ± 13.42
**REGRESSION STUDY**						
Chow	123.60 ± 11.02	1624.35 ± 970.91	103.22 ± 9.74	190.78 ± 41.59	1.57 ± 0.28	27.85 ± 10.78
Western_Baseline	132.90 ± 10.64	581.91 ± 102.04	358.33 ± 31.80	75.00 ± 21.48	28.14 ± 3.27	269.00 ± 23.84
Baseline_Western_42d	118.44 ± 16.74	1283.14 ± 1027.10	322.47 ± 54.37	64.07 ± 16.42	19.62 ± 3.73	304.93 ± 59.42
Baseline_Western _84d	122.04 ± 13.23	1652.76 ± 1077.47	346.07 ± 45.00	75.8 ± 15.74	24.91 ± 3.68	246.87 ± 25.39
Baseline_Western_112d	123.51 ± 17.13	637.17 ± 124.96	344.38 ± 56.47	71.69 ± 17.10	26.30 ± 4.22	268.85 ± 29.12
Baseline_Chow_42d	126.36 ± 9.46	1936.51 ± 824.14	157.00 ± 32.85	85.47 ± 28.85	4.05 ± 1.27	112.37 ± 45.13
Baseline_Chow_84d	127.20 ± 9.94	3577.69 ± 2746.06	158.20 ± 35.46	101.93 ± 35.69	3.95 ± 1.26	105.96 ± 47.88
Baseline_Chow_112d	N/A	N/A	123.81 ± 13.02	N/A	N/A	144.21 ± 34.69
**PHARMACOTHERAPY STUDY**						
Chow_Vehicle	135.28 ± 10.29	2265.79 ± 1720.79	125.83 ± 29.21	148.65 ± 50.90	1.81 ± 0.68	15.94 ± 5.74
Western_Vehicle	135.50 ± 16.65	2487.69 ± 2721.10	323.90 ± 46.36	102.16 ± 29.51	60.71 ± 13.26	573.64 ± 193.54
Western_OCA_20 mg/kg	131.16 ± 11.80	2149.64 ± 978.52	238.27 ± 40.63	93.07 ± 24.62	47.22 ± 11.57	548.53 ± 243.08
Western_OCA_40 mg/kg	135.00 ± 10.78	2425.66 ± 2083.95	180.07 ± 41.90	131.27 ± 73.99	50.99 ± 11.49	431.67 ± 157.33
Western_CVC_10 mg/kg	132.99 ± 13.71	2343.83 ± 1153.26	351.33 ± 39.26	46.23 ± 17.77	N/A	N/A
Western_CVC_50 mg/kg	132.25 ± 15.92	3897.61 ± 2391.77	334.57 ± 42.71	55.16 ± 18.06	N/A	N/A
**Group**	**ALT (U/L)**	**AST (U/L)**	**CK18 (μU/mL)**	**TIMP−1 (pg/mL)**	**PIIINP (ng/mL)**	**HA (pg/mL)**
**PROGRESSION STUDY**						
Chow_60d	41.47 ± 30.18	155.93 ± 114.81	223933.20 ± 25517.89	681.49 ± 105.24	31881.14 ± 3684.61	325.06 ± 112.20
Chow_150d	41.80 ± 8.83	72.50 ± 17.26	352317.23 ± 126257.52	565.66 ± 102.58	40008.05 ± 6247.07	358.17 ± 65.96
Chow_254d	25.80 ± 9.64	80.10 ± 43.92	N/A	965.15 ± 198.13	N/A	N/A
Chow_352d	26.70 ± 7.79	53.20 ± 14.82	N/A	1044.62 ± 119.28	N/A	N/A
Western_60d	131.18 ± 177.85	212.90 ± 263.06	302565.79 ± 67516.99	2367.45 ± 2489.28	32351.67 ± 10247.28	383.14 ± 276.84
Western_150d	164.56 ± 141.28	163.90 ± 89.76	578182.14 ± 140675.56	2049.92 ± 1082.22	51084.35 ± 10408.85	221.53 ± 93.25
Western_254d	331.64 ± 103.06	367.29 ± 98.74	N/A	6426.91 ± 1626.33	N/A	N/A
Western_352d	24.72 ± 4.52	268.71 ± 34.97	N/A	368.43 ± 134.07	N/A	N/A
**REGRESSION STUDY**						
Chow	32.89 ± 7.27	123.67 ± 72.38	402573.70 ± 143938.98	707.78 ± 76.26	19680.59 ± 3093.38	N/A
Western_Baseline	349.33 ± 95.55	405.50 ± 78.10	N/A	1544.25 ± 189.64	N/A	N/A
Baseline_Western_42d	461.33 ± 114.63	426.87 ± 105.69	866330.78 ± 137949.85	7001.57 ± 1492.63	34063.72 ± 4967.04	N/A
Baseline_Western _84d	450.93 ± 116.82	436.8 ± 113.54	846016.16 ± 136224.76	8570.57 ± 1468.49	32081.29 ± 3955.33	N/A
Baseline_Western _112d	339.08 ± 115.53	385.54 ± 109.08	N/A	7455.97 ± 2.20	N/A	N/A
Baseline_Chow_42d	78.93 ± 45.71	94.40 ± 32.66	514889.46 ± 96443.56	1584.27 ± 536.38	22204.10 ± 2610.13	N/A
Baseline_Chow_84d	73.87 ± 40.82	158.20 ± 69.88	472363.95 ± 130372.12	1764.76 ± 879.97	20529.14 ± 5439.83	N/A
Baseline_Chow_112d	3.21 ± 0.75	78.61 ± 38.30	N/A	127.57 ± 60.40	N/A	N/A
**PHARMACOTHERAPY STUDY**						
Chow_Vehicle	78.60 ± 85.16	119.10 ± 78.81	N/A	886.50 ± 181.05	N/A	N/A
Western_Vehicle	348.14 ± 84.15	345.21 ± 84.80	N/A	5584.61 ± 1265.30	N/A	N/A
Western_OCA_20 mg/kg	382.20 ± 128.32	330.47 ± 102.67	N/A	5567.39 ± 2076.81	N/A	N/A
Western_OCA_40 mg/kg	599.80 ± 272.05	364.60 ± 144.39	N/A	5756.24 ± 2179.90	N/A	N/A
Western_CVC_10 mg/kg	N/A	N/A	N/A	N/A	N/A	N/A
Western_CVC_50 mg/kg	N/A	N/A	N/A	N/A	N/A	N/A

### Regression of hepatic lesions in diet-induced NASH model after switch to chow diet

**Figure 4:**
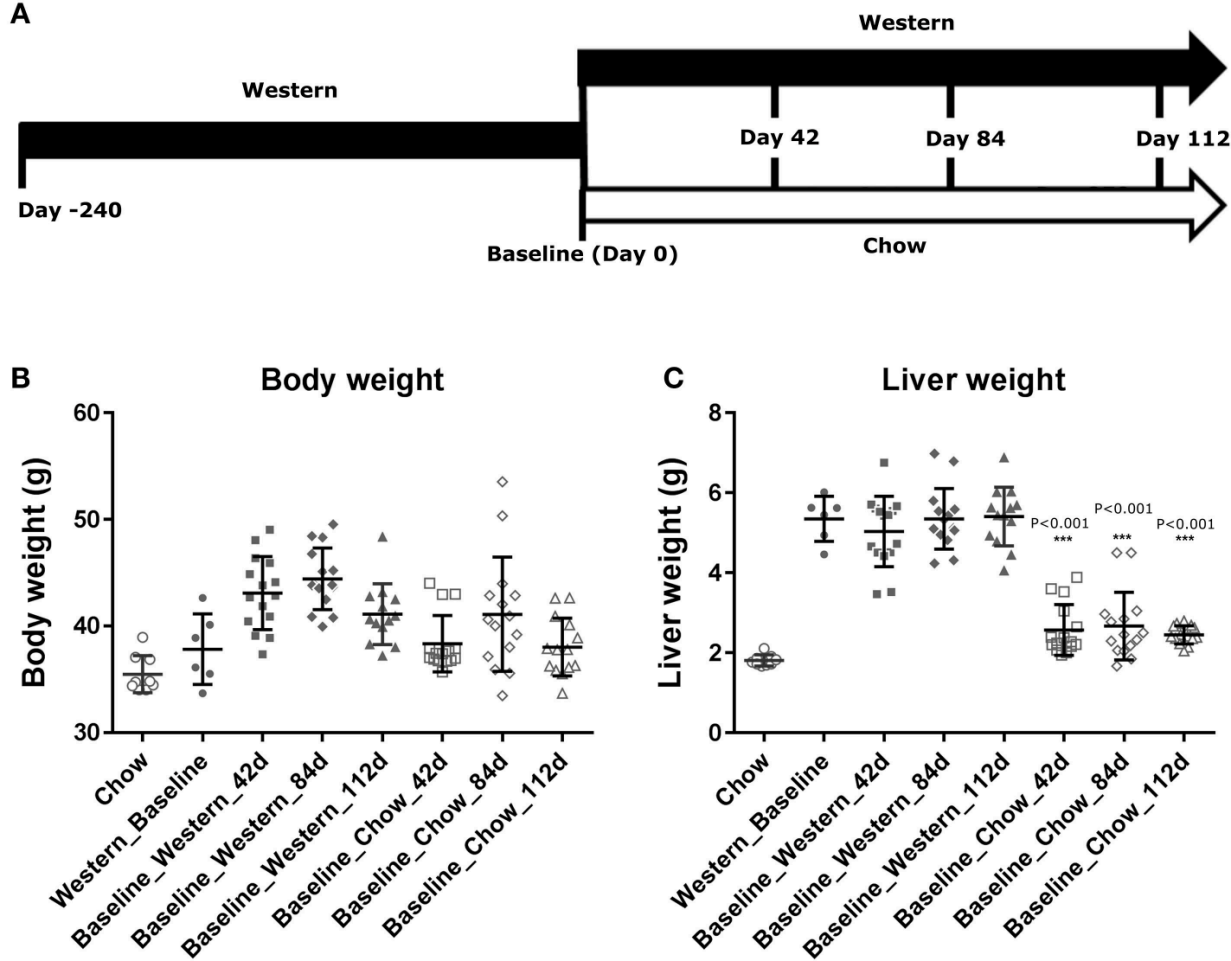
Changes in body and liver weight of the NASH mice after the Western diet was replaced by chow. Male C57BL/6J mice were fed Western diet for 240 days. The Western diet was then replaced by rodent chow as described in **(A)**. Their body weight **(B)** was recorded. Their liver weight **(C)** was measured immediately after euthanasia. The statistical analysis (One way ANOVA) was performed and statistical significance was indicated in the figure (*P* < 0.01, ***P < 0.001).

**Figure 5:**
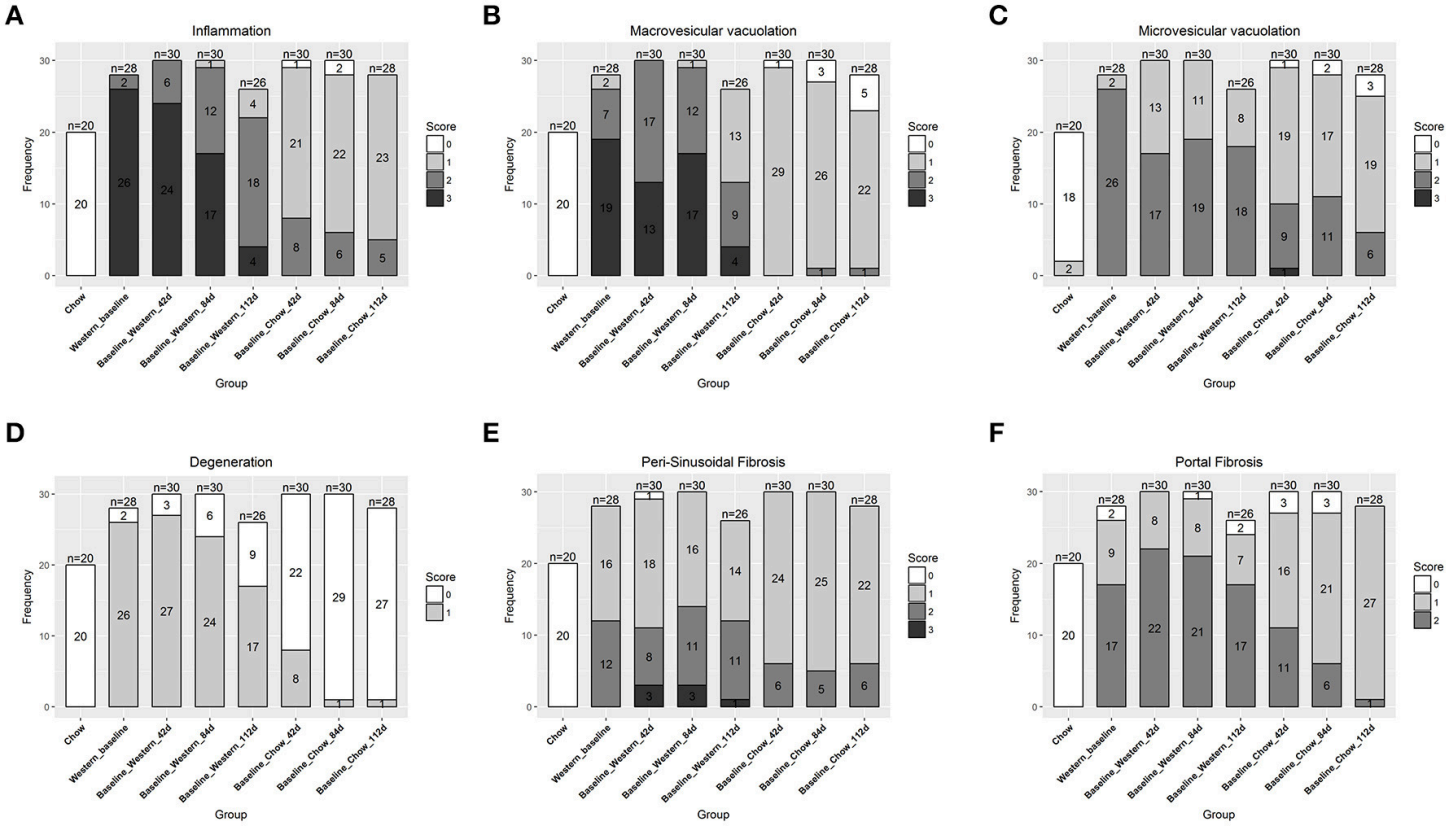
Regression of hepatic lesions in the NASH mice after the Western diet was replaced by chow. At different time after the dietary replacement, the right and left lateral lobes of the NASH mice were collected, and analyzed. The pathological scores from both lobes of an animal were pooled when performing categorical analysis. Hepatic inflammation **(A)**, macrovesicular vacuolation **(B)**, microvesicular vacuolation **(C)**, hepatocyte degeneration **(D)**, perisinusoidal fibrosis **(E)**, and portal fibrosis **(F)** were graded. Bridging fibrosis was also analyzed, but absent in all the animals.

**Figure 6:**
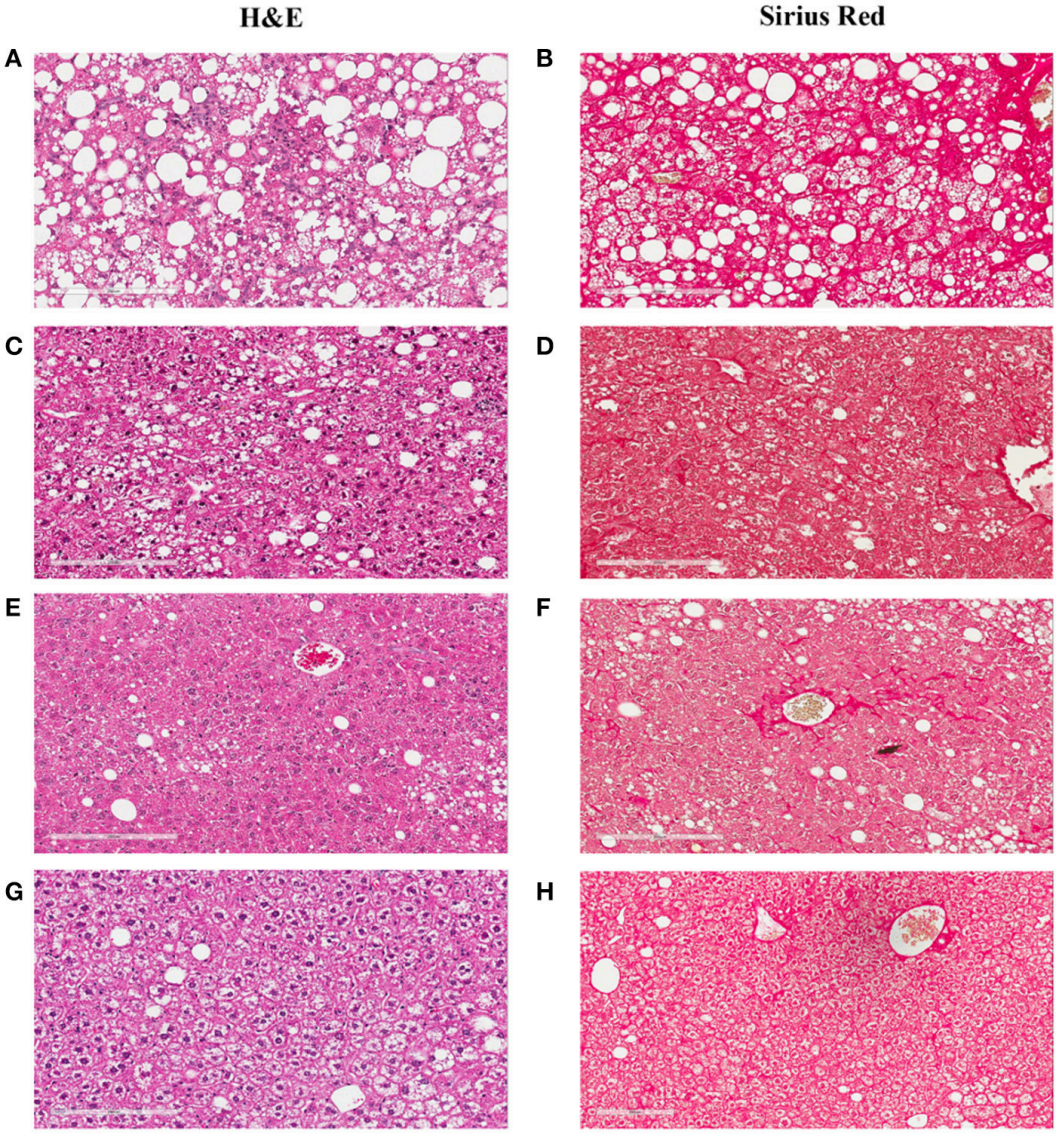
Hepatic lesion regression after the Western diet was replaced by chow. The representative images were taken from the liver sections of the NASH mice at baseline **(A,B)**, or after their Western diet was replaced by rodent chow for 42 **(C,D)**, 84 **(E,F)**, and 112 **(G,H)** days, respectively. **(A,C,E,G)** are the images of the liver sections stained with H&E; Panels **(B,D,F,H)** are the images of the liver sections stained with Sirius Red.

To assess the dynamics of lesion regression in the liver with established NASH, the mice were given Western diet for 240 days to establish baseline changes, then either continued on Western diet or switched to chow diet (Figure 4A). When the Western diet was replaced by chow diet, the liver weight decreased significantly, while the body weight was moderately reduced (Figures 4B,C) and hepatic steatosis decreased (Figures 5B,C, Table 3). Hepatic inflammation and hepatocyte degeneration were also greatly reduced (Figures 5A,D, Table 3). Portal fibrosis also decreased from day 42 to day 112 after diet replacement (Figures 5E,F, Table 3). Compared to mice continued on Western diet, mice switched to chow diet showed significant regression of all hepatic lesions at each of the time points, except for perisinusoidal fibrosis (adjust *P* value < 0.01, Table 4, Figures 6A–H; Supplementary Table 2). They also showed reduction of triglyceride and cholesterol levels in the liver, as well as ALT, AST, CK-18, TIMP-1, and PIIINP levels in the plasma (Table 2).

**Table 3:** Statistical analysis of regression study (all groups vs. Western_Baseline).

**Group**	**logOR**	**StdErr**	**AdjLower**	**AdjUpper**	**Adjp**
**INFLAMMATION**					
Baseline_Western_42d	1.1728	1.1479	−1.5846	3.9302	0.6160
Baseline_Western_84d	2.3234	1.0855	−0.2841	4.9308	0.0922
Baseline_Western_112d	4.3938	1.1145	1.7168	7.0709	0.0003
Baseline_Chow_42d	7.2772	1.2409	4.2964	10.2579	< 0.0001
Baseline_Chow_84d	7.7610	1.2954	4.6493	10.8727	< 0.0001
Baseline_Chow_112d	7.5749	1.2659	4.5341	10.6157	< 0.0001
**MACROVESICULAR VACUOLATION**					
Baseline_Western_42d	0.7883	0.6008	−0.7222	2.2988	0.5332
Baseline_Western_84d	0.3894	0.6422	−1.2252	2.0040	0.9628
Baseline_Western_112d	3.1289	0.7437	1.2592	4.9985	0.0001
Baseline_Chow_42d	6.7377	0.9389	4.3771	9.0982	< 0.0001
Baseline_Chow_84d	7.1056	1.2195	4.0397	10.1715	< 0.0001
Baseline_Chow_112d	7.7534	1.3235	4.4259	11.0809	< 0.0001
**MICROVESICULAR VACUOLATION**					
Baseline_Western_42d	2.0143	0.8456	−0.01284	4.0414	0.0519
Baseline_Western_84d	1.7488	0.8183	−0.2131	3.7107	0.0919
Baseline_Western_112d	1.4956	0.8360	−0.5086	3.4999	0.1877
Baseline_Chow_42d	2.9245	0.9613	0.6199	5.2290	0.0083
Baseline_Chow_84d	2.9303	0.8754	0.8316	5.0291	0.0031
Baseline_Chow_112d	3.7208	0.9772	1.3779	6.0636	0.0006
**DEGENERATION**					
Baseline_Western_42d	0.3677	1.1858	−2.5410	3.2765	0.9971
Baseline_Western_84d	1.1787	1.1105	−1.5453	3.9026	0.6474
Baseline_Western_112d	1.9290	1.1021	−0.7745	4.6324	0.2311
Baseline_Chow_42d	3.5766	1.1151	0.8412	6.3119	0.0056
Baseline_Chow_84d	5.9322	1.4407	2.3982	9.4663	0.0002
Baseline_Chow_112d	5.8608	1.4407	2.3269	9.3947	0.0002
**PERI–SINUSOIDAL FIBROSIS**					
Baseline_Western_42d	0.1684	0.5572	−1.2627	1.5994	0.9995
Baseline_Western_84d	−0.3136	0.4861	−1.5622	0.9349	0.9714
Baseline_Western_112d	−0.1823	0.4732	−1.3976	1.0329	0.9980
Baseline_Chow_42d	1.0093	0.5999	−0.5314	2.5501	0.3496
Baseline_Chow_84d	1.2108	0.5562	−0.2177	2.6392	0.1312
Baseline_Chow_112d	0.9296	0.6026	−0.6180	2.4772	0.4384
**PORTAL FIBROSIS**					
Baseline_Western_42d	−0.6791	0.6202	−2.2395	0.8812	0.6934
Baseline_Western_84d	−0.4707	0.6020	−1.9851	1.0437	0.8957
Baseline_Western_112d	−0.1919	0.6599	−1.8520	1.4682	0.9992
Baseline_Chow_42d	1.0247	0.6123	−0.5158	2.5652	0.3068
Baseline_Chow_84d	1.6957	0.6414	0.08216	3.3092	0.0356
Baseline_Chow_112d	1.9616	0.5782	0.5071	3.4162	0.0035

*log(OR) of all groups to Western_Baseline group calculated by Proportional Odds Model (Dunnett's adjustment for groups with OR estimates)*.

**Table 4:** Statistical analysis of regression study (Chow vs. Western).

**Group**	**logOR**	**StdErr**	**AdjLower**	**AdjUpper**	**Adjp**
**INFLAMMATION**					
Baseline_Chow_42d vs. Baseline_Western_42d	29.2607	0	N/A*	N/A*	N/A*
Baseline_Chow_84d vs. Baseline_Western _84d	5.0523	1.0834	2.9288	7.1758	< 0.0001
Baseline_Chow_112d vs. Baseline_Western _112d	3.2852	0.7856	1.7455	4.8249	< 0.0001
**MACROVESICULAR VACUOLATION**					
Baseline_Chow_42d vs. Baseline_Western_42d	57.3394	0	N/A*	N/A*	N/A*
Baseline_Chow_84d vs. Baseline_Western _84d	6.7856	1.3822	4.0764	9.4947	< 0.0001
Baseline_Chow_112d vs. Baseline_Western _112d	3.5085	0.9957	1.5570	5.4601	0.0004
**MICROVESICULAR VACUOLATION**					
Baseline_Chow_42d vs. Baseline_Western_42d	0.9167	0.5746	−0.2095	2.0430	0.1106
Baseline_Chow_84d vs. Baseline_Western _84d	1.1657	0.4404	0.3026	2.0288	0.0081
Baseline_Chow_112d vs. Baseline_Western _112d	2.1815	0.6481	0.9113	3.4518	0.0008
**DEGENERATION**					
Baseline_Chow_42d vs. Baseline_Western_42d	3.2088	0.7041	1.8288	4.5889	< 0.0001
Baseline_Chow_84d vs. Baseline_Western _84d	4.7536	1.0747	2.6471	6.8600	< 0.0001
Baseline_Chow_112d vs. Baseline_Western _112d	3.9318	1.0660	1.8425	6.0211	0.0002
**PERI–SINUSOIDAL FIBROSIS**					
Baseline_Chow_42d vs. Baseline_Western_42d	0.7572	0.5981	−0.4151	1.9295	0.2055
Baseline_Chow_84d vs. Baseline_Western _84d	1.5357	0.5175	0.5214	2.5500	0.0030
Baseline_Chow_112d vs. Baseline_Western _112d	1.1753	0.5606	0.07655	2.2740	0.0360
**PORTAL FIBROSIS**					
Baseline_Chow_42d vs. Baseline_Western_42d	1.6321	0.5340	0.5855	2.6787	0.0022
Baseline_Chow_84d vs. Baseline_Western _84d	2.1207	0.5450	1.0525	3.1888	< 0.0001
Baseline_Chow_112d vs. Baseline_Western _112d	2.7502	0.9053	0.9758	4.5246	0.0024

log(OR) of Chow groups to Western groups calculated by Proportional Odds Model (Dunnett's adjustment for groups with OR estimates). *Indicates N/A: all observations of a group equal to the same score so the odds can not be estimated. Proportional Odds Model is not applicable in such case.

### Effects of obeticholic acid and CCR2/5 antagonist on hepatic lesions of the NASH mouse model

**Figure 7:**
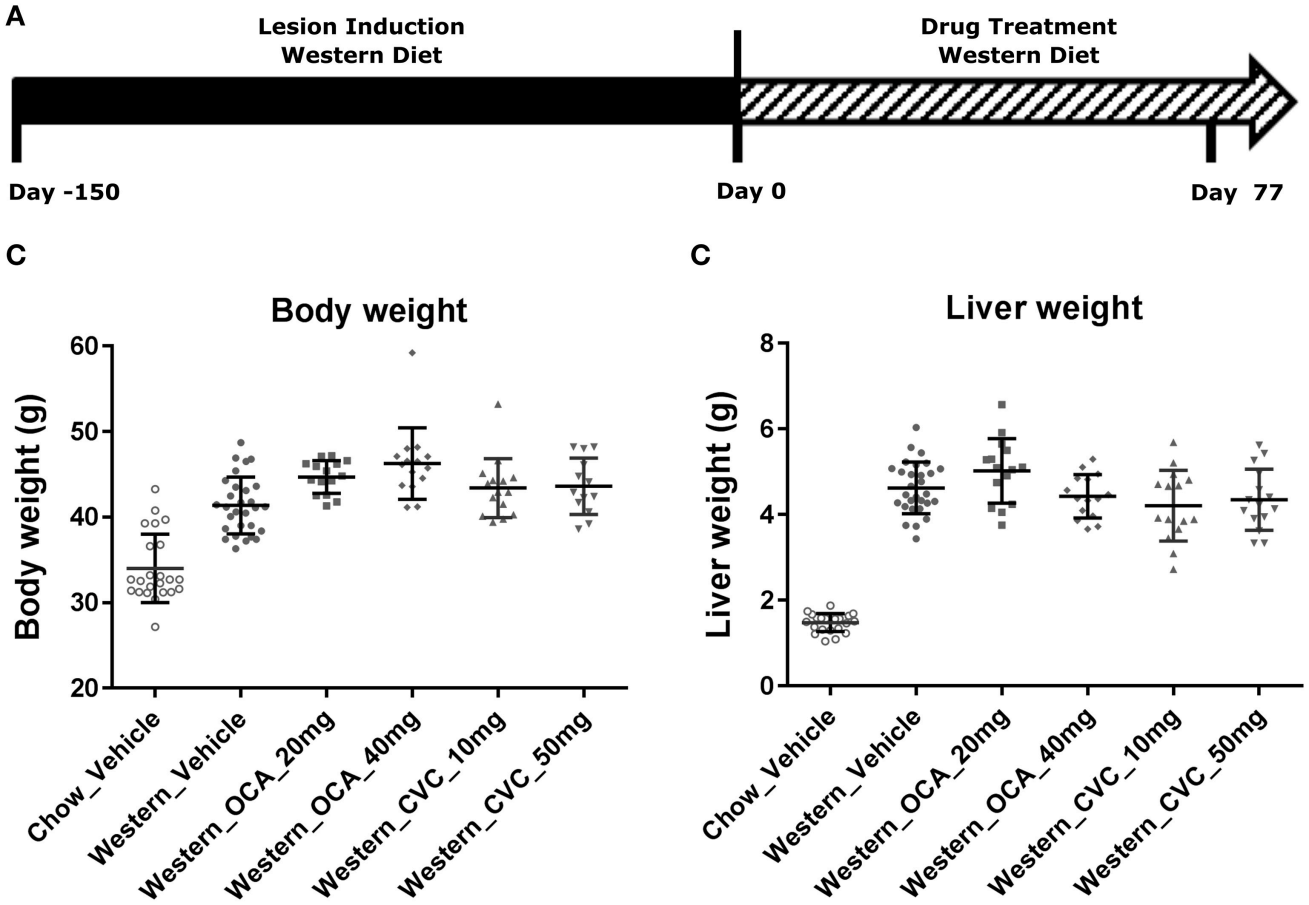
Pharmacological studies of Ocaliva (OCA) and Cenicriviroc (CVC) in the NASH Mice induced by Western diet. NASH mice fed Western diet for 150 days were treated with vehicle, OCA or CVC as described in **(A)**. Their body weight **(B)** recorded before euthanasia, and liver weight **(C)** were measured immediately after euthanasia. The statistical analysis (One way ANOVA) was performed and no statistical significance was found.

**Figure 8:**
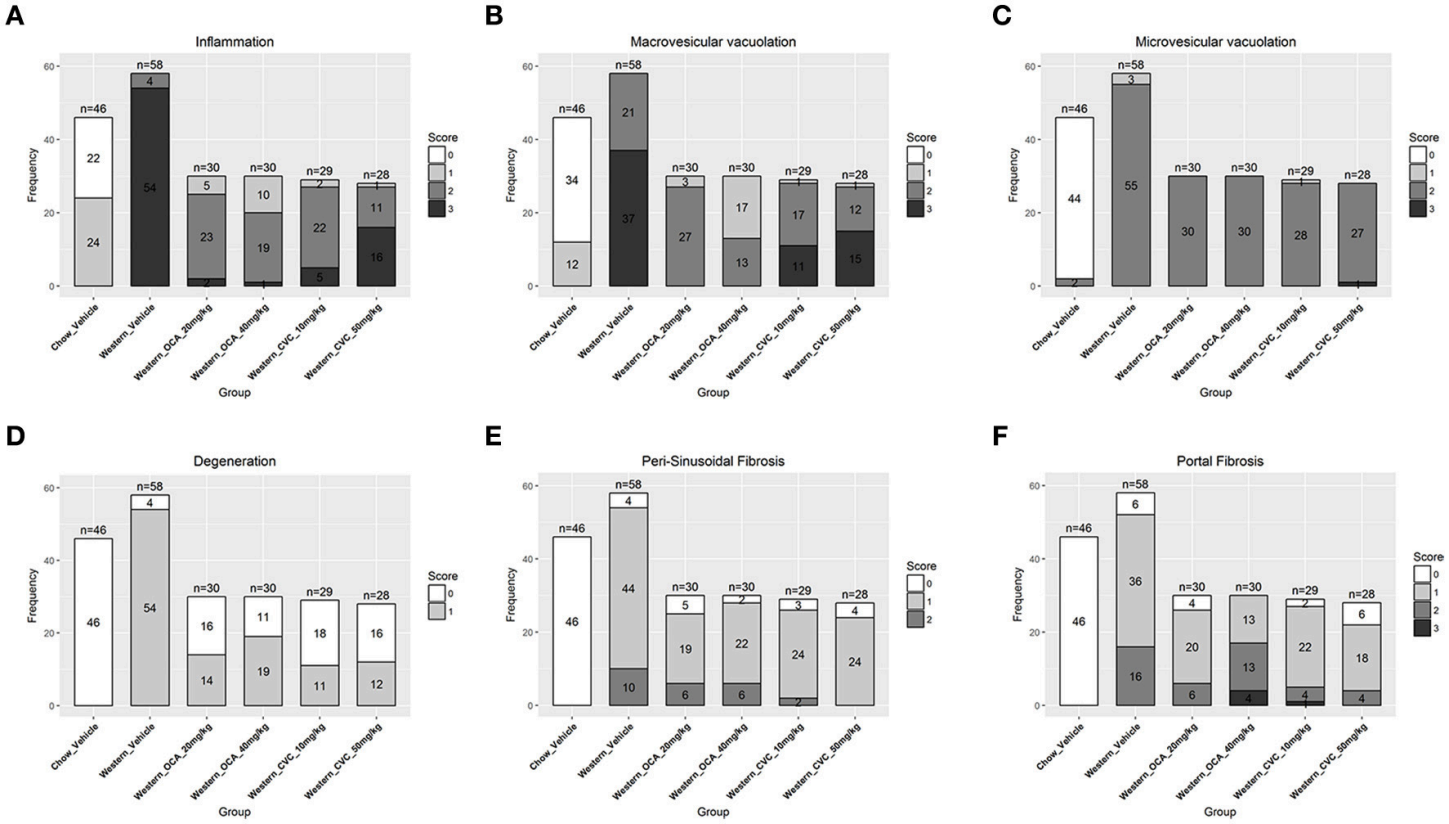
Hepatic lesions of the NASH MIce treated with Ocaliva (OCA) and Cenicriviroc (CVC). After 77 days of drug treatment, the right and left lateral lobes of the NASH mice were collected, and analyzed. The pathological scores from both lobes of an animal were pooled when performing categorical analysis. Hepatic inflammation **(A)**, macrovesicular vacuolation **(B)**, microvesicular vacuolation **(C)**, hepatocyte degeneration **(D)**, perisinusoidal fibrosis **(E)**, and portal fibrosis **(F)** were graded. Bridging fibrosis was also analyzed, but absent in all the animals.

**Figure 9:**
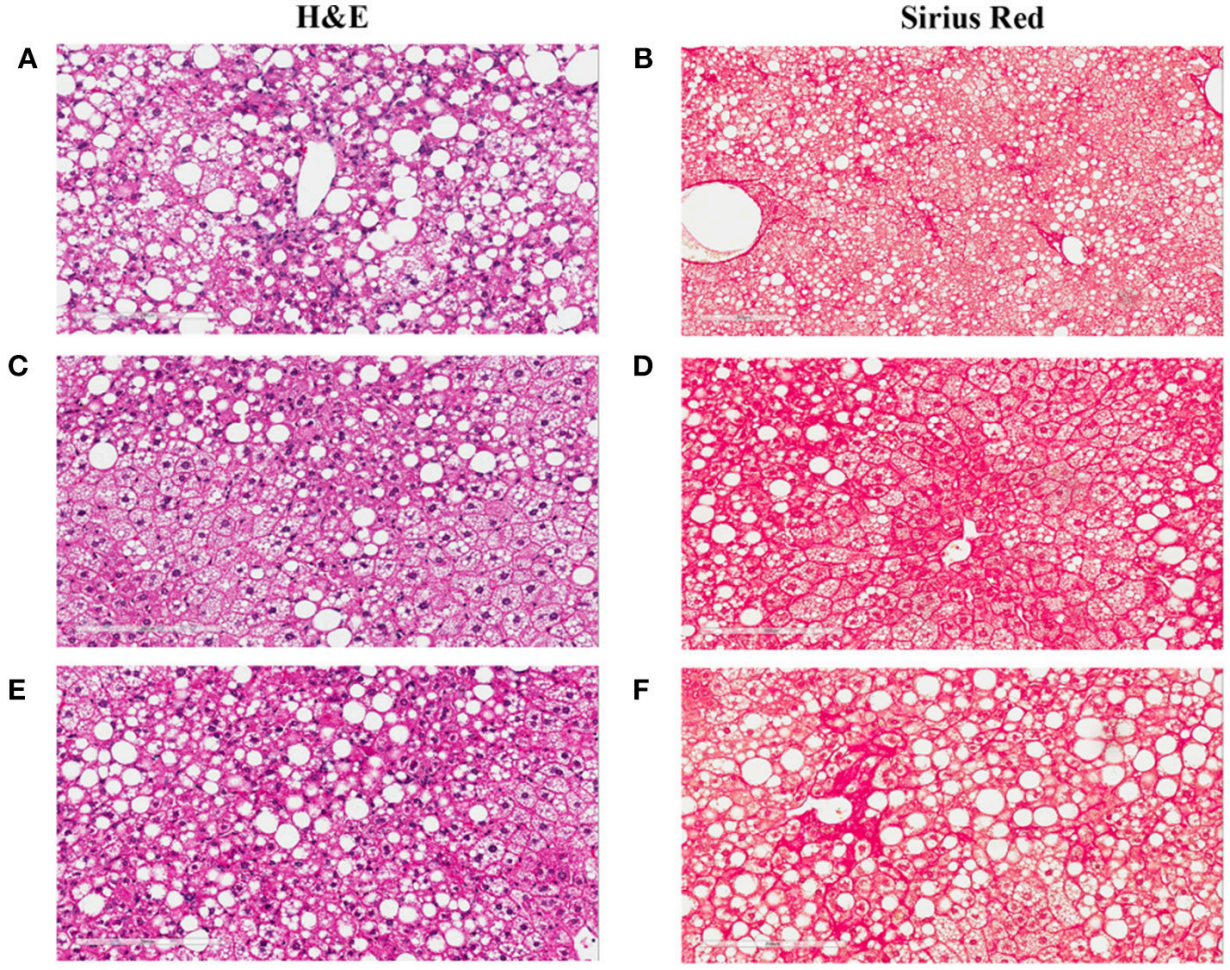
Liver images of the NASH mice treated with Ocaliva (OCA) and Cenicriviroc (CVC). The representative images were taken from the liver sections of the NASH mice treated with vehicle **(A,B)**, 20 mg/kg/day **(C,D)**, and 40 mg/kg/day **(E,F)** of OCA. **(A,C,E)** are the images of the liver sections stained with H&E; **(B,D,F)** are the images of the liver sections stained with Sirius Red.

To understand the effects of pharmacotherapy on hepatic lesions, mice with established NASH (150 days on Western diet) were treated with either obeticholic acid (OCA), or Cenicriviroc (CVC) for 11 weeks (Figure 7A). OCA or CVC treatment didn't alter the body weight or liver weight significantly (Figures 7B,C). At 20 and 40 mg/kg doses, OCA significantly reduced inflammation, macrovesicular vacuolation and hepatic degeneration (Figures 8A,B,D, Table 5). No improvement of perisinusoidal and portal fibrosis were observed with OCA treatment (Figures 8E,F, 9A–F). At 40 mg/kg dose of OCA treatment, moderate increase of portal fibrosis was observed (Figure 8F). In consistent with this observation, the ALT levels in mice treated with 40 mg/kg OCA were elevated (Table 2). At both 10 and 50 mg/kg doses, CVC decreased inflammation and hepatocyte degeneration (Figures 8A,D, Table 5). There was a moderate decrease of perisinusoidal fibrosis and there was no effect on hepatic steatosis and portal fibrosis (Figures 8B,C,E,F).

**Table 5:** Statistical analysis of pharmacotherapy study.

**Group**	**logOR**	**StdErr**	**AdjLower**	**AdjUpper**	**Adjp**
**INFLAMMATION**					
Western_OCA_20 mg/kg	5.0926	0.9093	2.9196	7.2656	< 0.0001
Western_OCA_40 mg/kg	5.9482	0.9591	3.6561	8.2403	< 0.0001
Western_CVC_10 mg/kg	4.1329	0.8807	2.0282	6.2377	< 0.0001
Western_CVC_50 mg/kg	2.3564	0.9136	0.1733	4.5395	0.0304
**MACROVESICULAR VACUOLATION (SIMULATE MULTIPLICITY ADJUSTMENT^#^)**					
Western_OCA_20 mg/kg	3.7925	0.7453	1.9543	5.6307	< 0.0001
Western_OCA_40 mg/kg	5.9303	1.0134	3.4308	8.4298	< 0.0001
Western_CVC_10 mg/kg	1.1297	0.6260	−0.4141	2.6736	0.2286
Western_CVC_50 mg/kg	0.4868	0.6118	−1.0221	1.9958	0.8649
**MICROVESICULAR VACUOLATION**					
Western_OCA_20mg/kg	−26.0316	1.0236	−28.3371	−23.7261	< 0.0001
Western_OCA_40mg/kg	−26.0316	1.0236	−28.3371	−23.7261	< 0.0001
Western_CVC_10mg/kg	−0.4235	1.2849	−3.3175	2.4706	0.9585
Western_CVC_50mg/kg	−51.7739	1.2849	−54.6679	−48.8798	< 0.0001
**DEGENERATION**					
Western_OCA_20 mg/kg	2.7362	0.8769	0.6430	4.8295	0.0060
Western_OCA_40 mg/kg	2.0561	0.8964	−0.08355	4.1958	0.0629
Western_CVC_10 mg/kg	3.0952	0.8825	0.9886	5.2017	0.0016
Western_CVC_50 mg/kg	2.8904	0.9103	0.7174	5.0634	0.0050
**PERI–SINUSOIDAL FIBROSIS**					
Western_OCA_20 mg/kg	0.3330	0.8115	−1.6704	2.3363	0.9867
Western_OCA_40 mg/kg	−0.1468	0.5475	−1.4985	1.2049	0.9974
Western_CVC_10 mg/kg	0.6871	0.5797	−0.7442	2.1183	0.6190
Western_CVC_50 mg/kg	1.1806	0.5189	−0.1005	2.4617	0.0824
**PORTAL FIBROSIS**					
Western_OCA_20 mg/kg	0.3941	0.5553	−0.9765	1.7646	0.9081
Western_OCA_40 mg/kg	−1.4945	0.4871	−2.6967	−0.2924	0.0083
Western_CVC_10 mg/kg	0.2432	0.4615	−0.8959	1.3822	0.9667
Western_CVC_50 mg/kg	0.9247	0.5742	−0.4926	2.3420	0.3334

*log(OR) of treatment groups to Western_Vehicle group calculated by Proportional Odds Model (Dunnett's adjustment for groups with OR estimates)*.

## Discussion

Metabolic disorders including dyslipidemia, insulin resistance, diabetes, and obesity are the known risk factors for NALFD and NASH (Dixon et al., 2001; Marchesini et al., 2003; Bedogni et al., 2005). NASH mice, induced by the Western diet (D0910301) develop hypercholesterolemia, insulin resistance and obesity. Therefore, they likely share, at least in part, etiology with human NASH patients. Biochemically, the NASH mice induced by the Western diet exhibit elevated ALT, AST, CK18, TIMP-1, and PIINP, the biomarkers indicative of NASH hepatic lesions in patients (Rosenberg et al., 2004). Microscopically, the NASH mice induced by the Western diet develop hepatic steatosis, necroinflammation and hepatocyte degeneration similar to ballooning in humans in a time-dependent manner. They also exhibit fibrosis in perisinusoidal (Zone 3) and portal areas. Overall, the NASH mice induced by the Western diet share important pathological and pathophysiological features with NASH patients, and should be a translatable animal model of human NASH.

Excessively consumption of several dietary components are implicated in NASH pathogenesis. Hepatic free fatty acids (FFAs) are markedly increased in NAFLD patients. Although most of FFAs accumulated in liver in the form of triglycerides are from lipolysis (Donnelly et al., 2005), dietary fat contribute to the increased adipose tissue in obese NAFLD patients, that could indirectly contribute to hepatic steatosis. Hepatic FFA accumulation is directly linked to increased oxidative stress and impaired mitochondrial function, both of which induce inflammation and hepatocyte apoptosis (Cheung and Sanyal, 2008). Hepatic cholesterol crystals, similar to cholesterol clefts in atherosclerotic plagues, are recently observed in the kupffer cells of NASH patients and mice, but absent in the kupffer cells of patients and mice with simple steatosis. This observation led to the hypothesis that cholesterol crystallization within the lipid droplets of hepatocytes and aggregation of Kupffer cells in crown-like structures around such droplets represent an important, novel mechanism for progression of simple steatosis to NASH (Ioannou et al., 2013). Fructose increases hepatic steatosis and oxidative stress via several mechanisms. Fructose increases hepatic *de novo* lipogenesis, enhances malonyl-CoA concentration, inhibits mitochondrial β-oxidation and decreases mitochondrial ATP production (Schmid et al., 2011). In addition, phosphorylation of fructose to fructose-1-phosphate leads to depletion of hepatic ATP and increase in ADP and inosine monophosphate (IMP), which is converted to uric acid (Nakagawa et al., 2006), that also promotes steatosis inducing mitochondrial oxidative stress (Lanaspa et al., 2012). Although the mechanisms for those dietary components caused hepatic lesions have been intensely studied, lesion regression and reversal have not been systematically studied. Besides the metabolic pathways, recent studies have shown that micro RNAs, such as miR-122, miR-192, miR-21, miR-29a, miR-34a, and miR-505, are up or down regulated at disease state of NASH and may play a role in the disease progression (Liu et al., 2018).

Understanding the regression and resolution of NASH hepatic lesions is critical for the development of an effective therapeutic strategy. In our diet-induced animal model of NASH, hepatic lesion regression was studied by replacing the NASH causing Western diet with regular rodent chow. The replacement of the NASH causing Western diet eliminated caused a time-dependent decrease in body weight, plasma cholesterol and insulin concentrations, indicative of correction of metabolic abnormalities in these animals. They also exhibited a time-dependent normalization/improvement in ALT and AST activities, and CK18, TIMP-1, and PNIIINP concentrations, suggesting regression or cessation of hepatic injury. Microscopically, macrovesicular vacuolations, dissipated after dietary intervention; inflammation and ballooning degeneration were also reduced over time. The results are consistent with existing reports of clinical trials, which demonstrated that life style modification (Vilar-Gomez et al., 2015) including calorie restriction, physical activity and body weight loss improve hepatic NASH lesions. Activation of hepatic stellate cells (HSC) is critical for hepatic fibrosis and TGF-beta plays the cardinal role in this process. HSC activation causes cell fate transition and proliferation of myofibroblast and production of ECM proteins during liver fibrosis. Upon activation of HSC, increased matrix production is closely correlated with an increase of TIMP-1 secretion from activated HSC. In fact, TIMP-1 and P3NP are one of the biomarker recognized by European Medicines Agency for diagnosis of NASH. Consistent with NASH patients, in the Western diet fed mice, we observed elevated levels of collagen fragments (P3NP) and TIMP-1 (shown in Table 2), which strongly suggests the activation of HSC and production of ECM protein in Western diet fed animals.

It has been reported that regression and resolution of fibrosis, rather than hepatic steatosis or inflammation, is correlated with reduction in mortality and liver transplantation in NASH patients (Angulo et al., 2015). In our lesion regression experiments, replacement of the NASH causing Western diet by rodent chow significantly slowed down the progression of perisinusoidal and portal fibrosis, but exhibited no significant effects on existing perisinusoidal fibrosis even after 112 days of dietary intervention. Due to anti-viral therapies successfully achieving regression of fibrosis and cirrhosis in HBV (Hepatitis B) and HCV (Hepatitis C) patients (van Zonneveld et al., 2006; George et al., 2009; Rockey, 2016), there is an optimism that a similar success could be repeated in NASH patients when the initial insults are removed as repair and remodeling processes are similar in chronic liver diseases. Increased evidence suggests that NASH pathogenesis, although sharing the remodeling processes with other chronic liver diseases, may have its unique characteristics. Kleiner et al. (Kleiner and Brunt, 2012; Kleiner and Makhlouf, 2016) recently reported that NASH fibrosis in adults exhibit a different pattern from that of viral hepatitis in that the fibrosis of NASH that begins in acinar zone 3 of perisinusoidal space and expands to portal areas, whereas the fibrosis of viral hepatitis usually starts in periportal areas (although both patterns could eventually lead to cirrhosis). This unique progression pattern of NASH fibrosis clearly contributes to the earlier, but slower development of NASH fibrosis relative to hepatitis B and C. We first demonstrated in preclinical model that perisinusoidal fibrosis of NASH is more difficult to treat by dietary intervention than portal fibrosis. This observation needs to be confirmed in NASH patients when effective pharmacotherapy is established.

To study the effects of current investigational drugs on NASH lesion progression and regression, we treated the diet-induced NASH mice with Ocaliva (OCA), an agonist of FXR and a metabolic modulator, and Cenicriviroc (CVC), an inhibitor of CCR2/5 which is considered as an anti-inflammatory and fibrotic agent. Ocaliva significantly decreased macrovesicular vacuolation, hepatocyte degeneration and necroinflammation in the NASH mice, but had no effect on perisinusoidal and portal fibrosis. When the dose of Ocaliva was increased to 40 mg/kg or higher, increased ALT and AST activities and portal fibrosis were observed. This was in contrast to the mild, but significant reduction in liver fibrosis reported in NASH patients in the Farnesoid X Receptor Ligand Obeticholic Acid in Non-alcoholic Steatohepatitis Treatment (FLINT) trial (Neuschwander-Tetri et al., 2015). However, recent updates on the clinical studies of Ocaliva demonstrated that this diet-induced animal model accurately recapitulated not only the efficacy, but also the adverse effects of Ocaliva in patients. First, a phase 2 trial of Ocaliva in Japan which shares a similar design with FLINT failed to confirm the anti-fibrotic effect of Oclava in NASH patients (Intercept Press Release Oct. 28, 2015). Second, United States Food and Drug Administration (FDA) recently issued a warning of serious hepatic injury caused by taking Ocaliva 5 mg daily, instead of 10 mg twice weekly as on the label, and consequently led to the death of 19 primary biliary cholangitis (PBC) patients (FDA, Drug Safety Communication Sept. 21, 2017). In our diet-induced NASH model CVC did not improve hepatic steatosis, but decreased inflammation and hepatocyte degeneration. In addition, CVC moderately decreased perisinusoidal fibrosis. Since NASH fibrosis has its own characteristics and removal of metabolic disorders may not be able to decrease or eliminate fibrosis in a timely manner, we propose that a combination of drug therapies that targets different pathogenic processes in NASH may be required for the NASH patients with advanced fibrosis. Metabolic modulators may only be effective in ameliorating steatosis, necroinflammation, and ballooning, which could thereby slow down the progression to fibrosis. An anti-fibrosis therapy is likely then required for treating existing fibrosis or cirrhosis.

## Author contributions

Z-MD: Conceived and designed the experiments. YX, HZ, SY, YS, JX, and HW: Performed the experiments. Z-MD, HZ, XW, HW, AU, and HH: Analyzed the data. Z-MD, XW, HW, AU, and HH: Wrote the paper.

### Conflict of interest statement

All authors are or were employees and may hold stock in Eli Lilly and Co. Eli Lilly and Co. provided support in the form of salaries for authors and funding for research materials, but did not have any additional role in the study design, data collection and analysis, decision to publish, or preparation of the manuscript. All relevant data are within the paper.
