# Corrigendum: Progression and Regression of Hepatic Lesions in a Mouse Model of NASH Induced by Dietary Intervention and Its Implications in Pharmacotherapy

**DOI:** 10.3389/fphar.2020.00093

**Published:** 2020-02-19

**Authors:** Zhi-Ming Ding, Yue Xiao, Xikun Wu, Haixia Zou, Shurong Yang, Yiyun Shen, Juehua Xu, Heather C. Workman, Amy L. Usborne, Haiqing Hua

**Affiliations:** ^1^ Lilly China R&D Center, Shanghai, China; ^2^ Covance Inc., Indianapolis, IN, United States; ^3^ Lilly Research Laboratories, Eli Lilly and Co., Indianapolis, IN, United States

**Keywords:** non-alcoholic steatohepatitis, steatosis, inflammation, fibrosis, obeticholic acid, CCR2/5, pathogenesis

In the original article, there was a mistake in **Figure 3D** as published. **Figure 3D** should represent a liver section from mice fed on a western diet for 60 days; however, a liver section from mice fed on chow diet was mistakenly used. The corrected **Figure 3** appears below.

**Figure 3:**
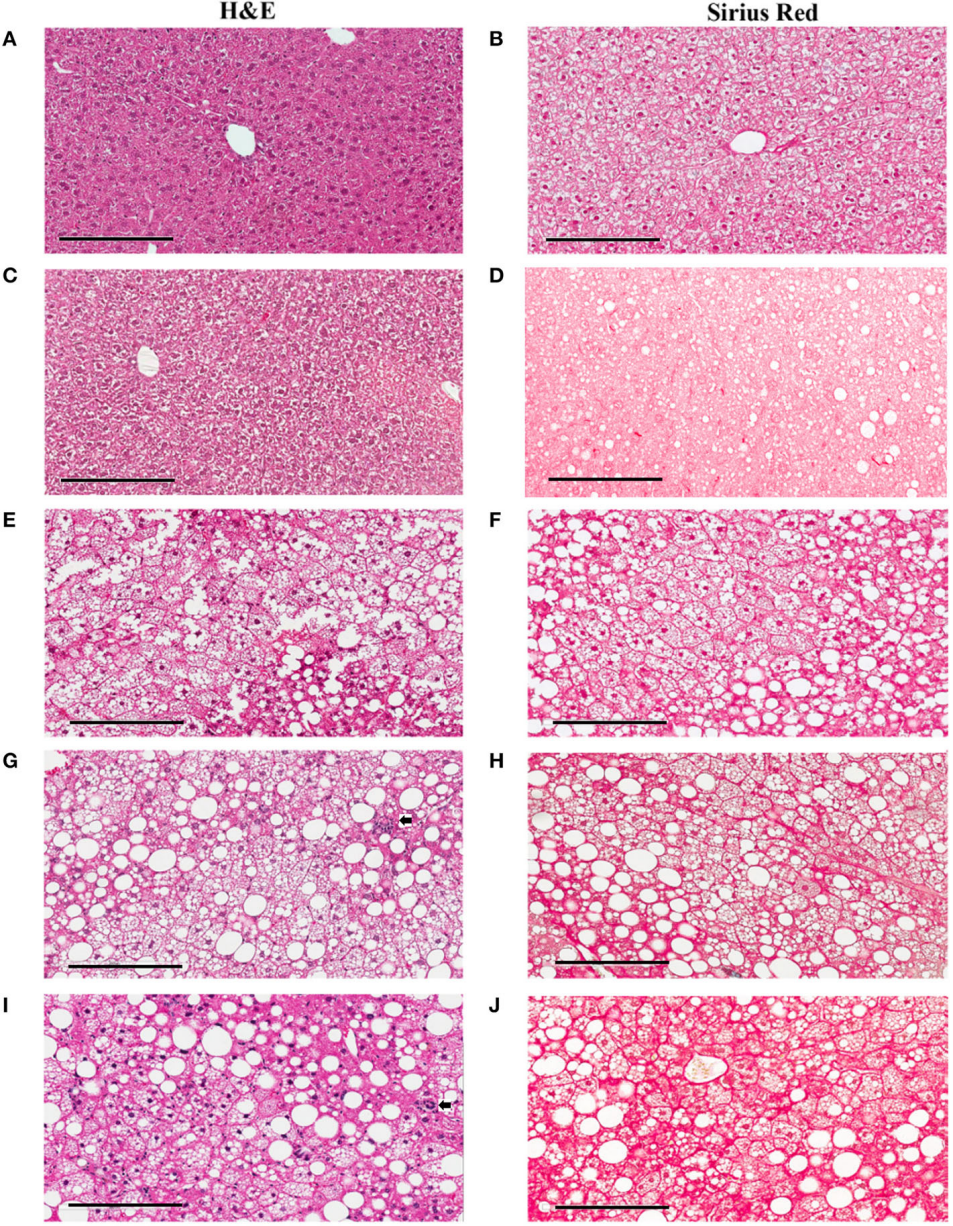
Hepatic lesions of the NASH Mice at Different Age. The representative images were taken from the liver sections of the mice fed either rodent chow **(A**, **B)** or Western diet for 60 **(C**, **D)**, 150 **(E**, **F)** and 245 **(G**, **H)**, and 352 **(I, J)** days respectively. **(A, C, E, G, I)** are the images of the liver sections stained with **H**&**E**; Panels **(B, D, F, H, J)** are the images of the liver sections stained with Sirius Red.

The authors apologize for this error and state that this does not change the scientific conclusions of the article in any way. The original article has been updated. 

