# Supplementary material for: Progression and Regression of Hepatic Lesions in a Mouse Model of NASH Induced by Dietary Intervention and Its Implications in Pharmacotherapy
Source: Front Pharmacol. 2018 May 1;9:410. doi: 10.3389/fphar.2018.00410 (PMC5938379; doi:10.3389/fphar.2018.00410)
Supplement: Supplementary file 1 [file Table_1.DOCX]

**Supplementary Table 1**

|  | Chow_60 | Chow_150 | Chow_254 | Chow_352 | Western_60 | Western_150 | Western_254 | Western_352 |
| --- | --- | --- | --- | --- | --- | --- | --- | --- |
| Inflammation | 0.0 | 0.0 | 0.0 | 0.0 | 22.5 | 47.5 | 100.0 | 100.0 |
| Macrovesicular vacuolation | 0.0 | 0.0 | 0.0 | 0.0 | 35.0 | 85.0 | 92.9 | 100.0 |
| Microvesicular vacuolation | 0.0 | 0.0 | 0.0 | 0.0 | 75.0 | 67.5 | 92.9 | 100.0 |
| Degeneration | 0.0 | 0.0 | 0.0 | 0.0 | 25.0 | 0.0 | 0.0 | 0.0 |
| Peri-Sinusoidal Fibrosis | 0.0 | 0.0 | 0.0 | 0.0 | 15.0 | 15.0 | 42.9 | 61.5 |
| Portal Fibrosis | 0.0 | 0.0 | 0.0 | 0.0 | 15.0 | 0.0 | 60.7 | 69.2 |

Percentage of animals with Score ≥2
