# Supplementary material for: Progression and Regression of Hepatic Lesions in a Mouse Model of NASH Induced by Dietary Intervention and Its Implications in Pharmacotherapy
Source: Front Pharmacol. 2018 May 1;9:410. doi: 10.3389/fphar.2018.00410 (PMC5938379; doi:10.3389/fphar.2018.00410)
Supplement: Supplementary file 2 [file Table_2.DOCX]

**Supplementary Table 2**

Percentage of animals with Score ≥2

|  | Chow | Western  _Baseline | Western  _42d | Western  _84d | Western  _112d | Chow  _42d | Chow  _84d | Chow  _112d |
| --- | --- | --- | --- | --- | --- | --- | --- | --- |
| Inflammation | 0.0 | 100 | 100 | 96.67 | 84.61 | 26.67 | 20 | 17.86 |
| Macrovesicular vacuolation | 0.0 | 92.86 | 100 | 96.67 | 50 | 0 | 3.33 | 3.57 |
| Microvesicular vacuolation | 0.0 | 92.86 | 56.67 | 63.33 | 69.23 | 33.33 | 36.67 | 21.43 |
| Degeneration | 0.0 | 0.0 | 0.0 | 0.0 | 0.0 | 0.0 | 0.0 | 0.0 |
| Peri-Sinusoidal Fibrosis | 0.0 | 42.86 | 40 | 36.67 | 46.15 | 20 | 16.67 | 21.42 |
| Portal Fibrosis | 0.0 | 50 | 73.33 | 70 | 65.38 | 36.67 | 30 | 3.57 |
