# Supplementary material for: Progression and Regression of Hepatic Lesions in a Mouse Model of NASH Induced by Dietary Intervention and Its Implications in Pharmacotherapy
Source: Front Pharmacol. 2018 May 1;9:410. doi: 10.3389/fphar.2018.00410 (PMC5938379; doi:10.3389/fphar.2018.00410)
Supplement: Supplementary file 3 [file Image_1.PDF]

Supplementary Figure 1

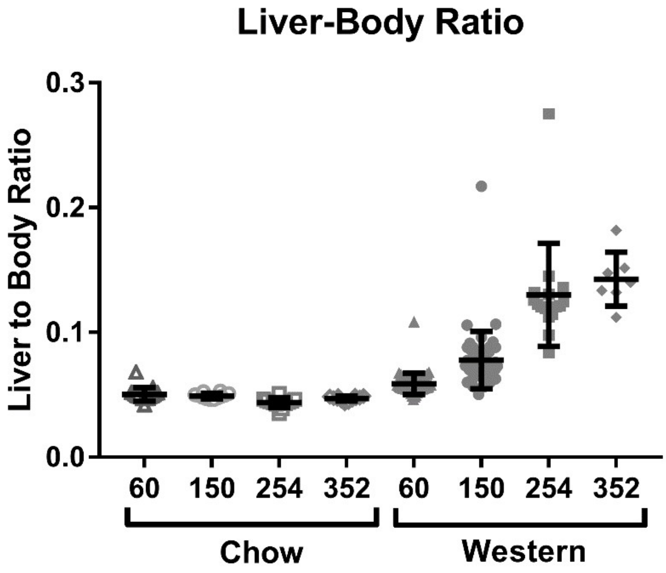

Changes in Liver to Body Weight Ratio after the Treatment with Western Diet

Male C57BL/6J mice were fed western diet as described in panel A of Figure 1. Their liver to body weight ratio was calculated.
