# Supplementary material for: Progression and Regression of Hepatic Lesions in a Mouse Model of NASH Induced by Dietary Intervention and Its Implications in Pharmacotherapy
Source: Front Pharmacol. 2018 May 1;9:410. doi: 10.3389/fphar.2018.00410 (PMC5938379; doi:10.3389/fphar.2018.00410)
Supplement: Supplementary file 4 [file Image_2.PDF]

Supplementary Figure 2

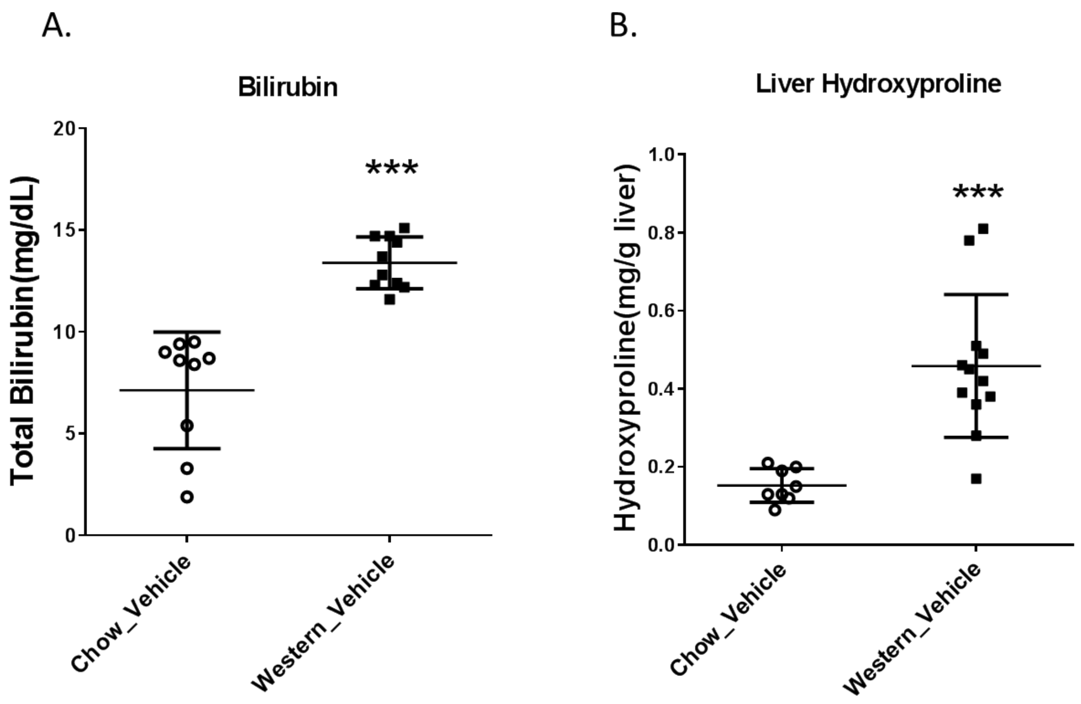

Plasma bilirubin and liver hydroxyproline between Chow diet and Western diet. Both increased significantly in Western diet condition compared to Chow diet control.

Male C57BL/6J mice were fed western diet as described in panel A of Figure 1 for 150 days. ***P<0.001 by t-test in GraphPad Prism.
